# A Rare Case of Microscopic Polyangitis With Associated Transient Hypophysitis

**DOI:** 10.1210/jcemcr/luaf010

**Published:** 2025-02-13

**Authors:** Mercedes Martinez-Gil, Monica Rodriguez, Jeremy Hughes, Savitha B Kalya, Kevin C J Yuen

**Affiliations:** Department of Internal Medicine, Creighton University School of Medicine, Phoenix, AZ 85013, USA; Barrow Pituitary Center, Barrow Neurological Institute, University of Arizona College of Medicine and Creighton University School of Medicine, Phoenix, AZ 85013, USA; Department of Neuroradiology, Barrow Neurological Institute, St. Joseph’s Hospital and Medical Center, Phoenix, AZ 85013, USA; Department of Rheumatology, Valleywise Comprehensive Health Center, Phoenix, AZ 85008, USA; Barrow Pituitary Center, Barrow Neurological Institute, University of Arizona College of Medicine and Creighton University School of Medicine, Phoenix, AZ 85013, USA

**Keywords:** granolomatosis with polyangitis, microscopic polyangitis, ANCA-associates vasculitides, pituitary enlargement

## Abstract

Granulomatosis with polyangiitis (GPA) and microscopic polyangiitis (MPA) are rare forms of antineutrophil cytoplasmic antibody-associated vasculitides, characterized by systemic inflammation and necrosis of small blood vessels, which can lead to multiorgan damage. GPA is identified by the presence of granulomas and serine proteinase-3 positivity, while MPA is marked by necrotizing vasculitis without granulomas and is associated with myeloperoxidase (MPO) positivity. Central nervous system involvement is more frequent in GPA, affecting 7% to 10% of patients, compared to its occurrence in approximately 1% of MPA cases. In this case report, we present a 41-year-old woman who exhibited symptoms of pituitary mass effect, initially suspected to be a macroadenoma. Further investigation revealed pituitary enlargement due to MPA, confirmed by positive MPO antibodies and lung biopsy findings consistent with MPA. After treatment with prednisone and rituximab, the patient’s pituitary gland returned to its normal size, with significant improvement in her symptoms. This case highlights the diagnostic complexities in differentiating MPA from more common causes of pituitary enlargement and underscores the necessity of considering vasculitic origins in similar clinical scenarios. Further research is essential to deepen the understanding of the pathophysiology and to optimize the management of pituitary involvement in MPA.

## Introduction

Granulomatosis with polyangiitis (GPA) and microscopic polyangiitis (MPA) are idiopathic antineutrophilic cytoplasmic antibody (ANCA)-associated vasculitides that cause inflammation and necrosis of the small blood vessels, leading to downstream ischemia and organ damage. The difference between GPA and MPA is that GPA is associated with the presence of granulomas on pathology, serine proteinase-3 positivity, higher frequency of sinonasal and upper airway involvement, and higher relapse rates. Conversely, MPA is characterized by systemic necrotizing vasculitis that pathologically demonstrates inflammation and necrosis of small-caliber blood vessels and occasionally medium-sized arteries without granulomas and with myeloperoxidase (MPO) positivity [1]. Both GPA and MPA can affect organ systems other than the airways and kidneys, but the involvement of the central nervous system (pituitary gland, meninges, and cerebral vasculature) is mainly observed in GPA, affecting between 7% and 11% of GPA patients [2, 3].

In contrast, pituitary involvement in MPA is exceedingly rare, with no incidence estimates currently available in the literature [4, 5]. Autoimmune vasculitis causes granulomatous inflammation, which when affecting the pituitary can cause pituitary mass effect resulting in headache, visual disturbance, as well as anterior and posterior pituitary hormone deficiencies [6]. Due to the rarity of pituitary dysfunction in MPA, the clinical manifestations of hypophysitis in MPA are not well defined in the existing medical literature. Furthermore, there are no specific treatment protocols directed to MPA-induced pituitary dysfunction, and treatment described usually involves immunosuppressive therapy aimed at the underlying condition [1].

In this case report, we describe for the first time a patient who presented with pituitary mass effect symptoms suggestive of an underlying pituitary macroadenoma, yet subsequent investigations revealed MPA-induced pituitary gland enlargement and, following the initiation of immunotherapy, the size of pituitary gland regressed back to its normal size.

## Case Presentation

**Figure 1.**
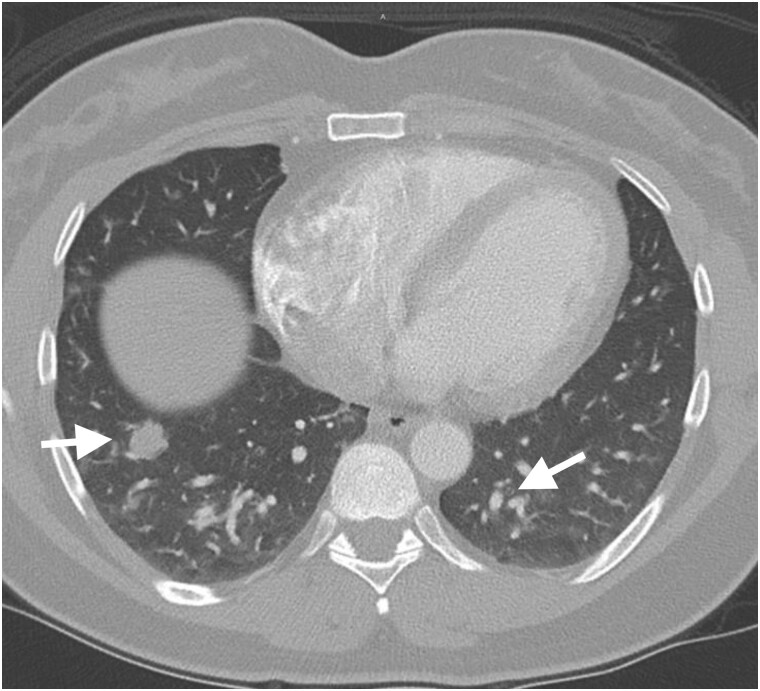
Contrast-enhanced chest computed tomography in lung window demonstrates multiple pulmonary nodules and mass lesions in the lungs bilaterally. Radiographic differential considerations would include both neoplastic and infectious etiologies.

**Figure 2.**
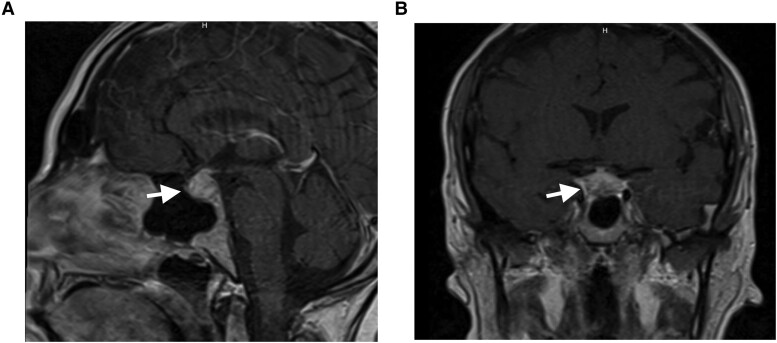
Postcontrast T1 weighted imaging in the sagittal (A) and coronal (B) planes demonstrates an enlarged heterogeneous pituitary gland measuring 1.7 × 1.2 × 1.3 cm with thickening of the infundibulum. There is no extension into the cavernous sinuses or compression of the optic chiasm.

A 41-year-old woman without any notable medical history presented to the emergency department due to ongoing headache, shortness of breath, and chest pains. The patient denied any polydipsia or polyuria. Her headache, which began as a sinus/facial discomfort 2 weeks prior and later extended to the back of her head, had persisted despite multiple consultations with ear, nose, and throat specialists, who subsequently excluded a sinus infection. Due to worsening of her symptoms, she was admitted to the hospital, and further investigations revealed elevated troponin levels (initially 0.109 ng/mL [109 ng/L], then 0.111 ng/mL [111 ng/L]; normal reference range: <0.01 ng/mL [<10 ng/L]), although an electrocardiogram showed no ischemic changes. A computed tomography angiogram of the chest revealed multiple pulmonary nodules and mass lesions, raising the possibility of potential metastatic disease, benign pulmonary nodules, or a pulmonary infection (Fig. 1). The patient's renal parameters were within normal limits. Because of her headache, pituitary-dedicated magnetic resonance imaging (MRI) was performed that revealed an enlarged heterogeneous pituitary gland measuring 1.7 × 1.2 × 1.3 cm with infundibular thickening but without invasion into the cavernous sinus and exerted no compression on the optic chiasm (Fig. 2). She was treated symptomatically for her headaches with topiramate, nortriptyline, and a 3-day course of dexamethasone, which slightly improved her symptoms, and she was discharged from the hospital after 3 days.

## Diagnostic Assessment

The patient was seen at the endocrinology outpatient clinic 1 week after hospital discharge, and pituitary hormone testing, including ACTH, morning cortisol, TSH, free T4, LH, FSH, prolactin, and IGF-I levels, were all within normal limits. Rheumatologic assessments identified antinuclear antibodies of 1:160 and a positive MPO/+P-ANCA. Inflammatory markers including C-reactive protein and erythrocyte sedimentation rate were elevated at 45 mg/L (4.5 mg/dL) (normal reference range: <10 mg/L [<1.0 mg/dL]) and 84 mm/hour (normal reference range: <20 mm/hour), respectively, while SSA and SSB antibodies, proteinase-3, and C-ANCA were all negative and C3 and C4 complement levels were within the normal range. A left upper lung biopsy identified reactive mesothelial tissue, mixed inflammation, focal necrosis, and occasional foreign body giant cells. Tuberculosis, HIV, hepatitis, and other infectious etiologies were subsequently ruled out. Based on these findings, the diagnosis of MPA was made.

The patient denied any family history of autoimmune diseases and was unaware of any medical history in her biological father's family. She also denied any oral or nasal ulcers, photosensitive rashes, alopecia, uncontrolled hypertension, miscarriages, and thrombo-embolic episodes. Additionally, the patient denied any history of allergic reactions, asthma, and illicit drug use and was not taking any medications. On further questioning, the patient revealed that she had lost 5 kg in weight in the previous month and had noticed worsening joint pain, dry mouth and some difficulty swallowing solids.

## Treatment

The patient was started on an initial dose of 60 mg a day of prednisone, and the dose was then tapered down by 10 mg a day dose decrements every 2 weeks, and she completed a course of glucocorticoid therapy over a total of 19 weeks. The patient also received 2 infusions of rituximab, spaced 2 weeks apart, and was scheduled to continue with these infusions every 6 months.

## Outcome and Follow-up

**Figure 3.**
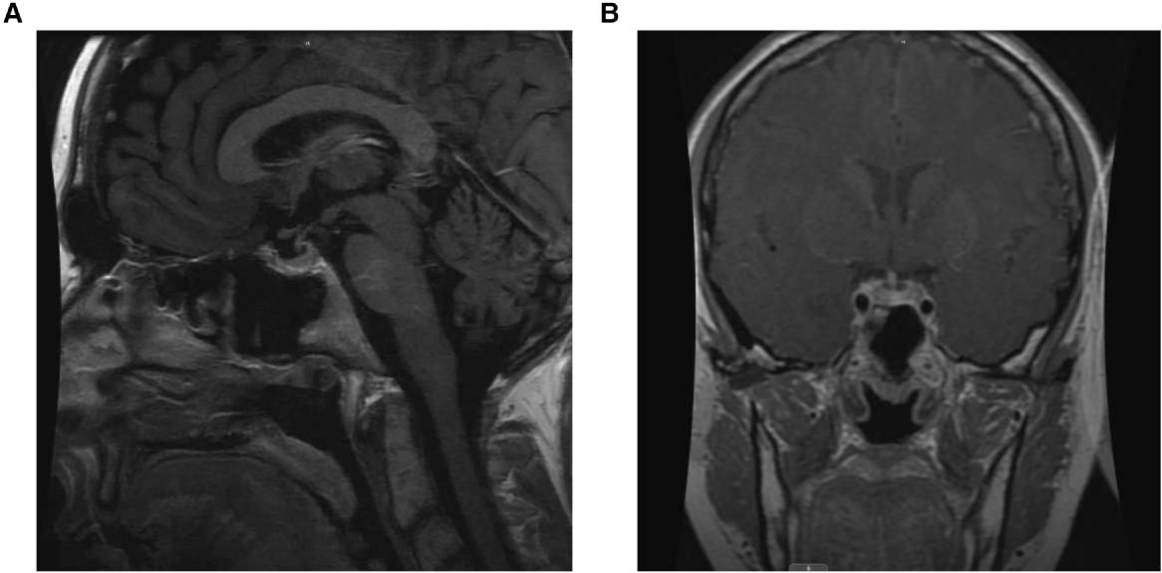
Postcontrast T1 weighted imaging in the sagittal (A) and coronal (B) planes demonstrates an interval decrease in the size of the pituitary gland and resolution of the previously observed infundibular thickening.

**Figure 4.**
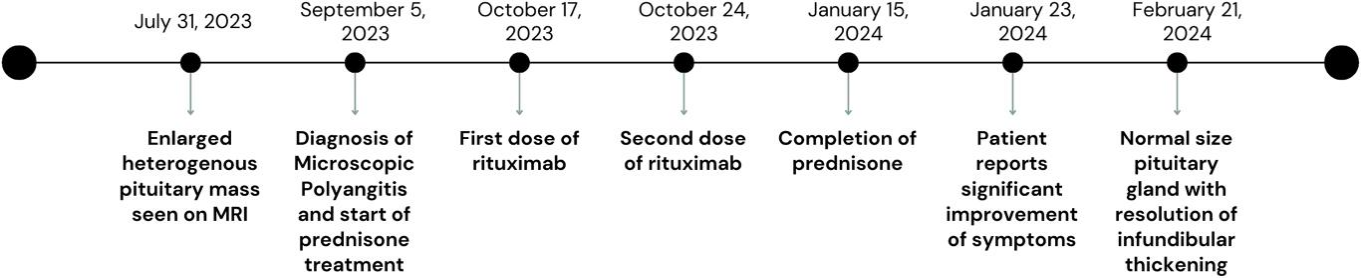
Chronological overview of clinical events.

Five months after prednisone treatment initiation, repeat MRI demonstrated that the size of the pituitary gland had regressed back to its normal size (Fig. 3) with the patient noting significant improvement on the frequency and severity of her headaches and joint pains 1 month after prednisone treatment initiation. The sequence of clinical events is displayed in Fig. 4.

## Discussion

Pituitary involvement in MPA is an exceedingly rare manifestation of this autoimmune vasculitis [4, 7]. Although its prevalence in MPA is undocumented, studies on GPA—a closely related condition—report pituitary dysfunction in approximately 1% of cases [4] In patients with GPA and pituitary involvement, common presentations include symptoms from mass effect and polyuria and polydipsia due to arginine vasopressin deficiency [4]. Despite treatment with glucocorticoids and immunosuppressive agents such as cyclophosphamide, rituximab, and methotrexate, pituitary dysfunction often persists, necessitating long-term hormone replacement therapy [8].

Conversely, the diagnosis of pituitary enlargement in our patient was made incidentally during MRI imaging for headache workup, which initially presented as sinus/facial discomfort. It is highly possible that if our patient did not report any headaches, MRI imaging would not have been performed and the diagnosis of pituitary enlargement would not have been made. The patient did not exhibit any symptoms to suggest underlying hypopituitarism, and follow-up MRI showed resolution of the pituitary enlargement with preservation of pituitary function and no evidence of structural damage to the gland after receiving treatment with prednisone and rituximab.

Our patient's initial MRI revealed relatively homogeneous enlargement of the pituitary gland, a finding that is nonspecific and can be found in patients presenting with pituitary adenoma; however, this observation can also be seen with infectious or inflammatory etiolgies as well as in normal young individuals and in pregnant women. Notably, the MRI demonstrated thickening of the infundibulum, which carries differential considerations including hypophysitis, neurosarcoidosis, and eosinophilic granuloma, as well as neoplastic etiologies including lymphoproliferative processes [9]. To differentiate hypophysitis from pituitary adenoma, 2 scoring systems have been described in the literature. Gutenberg et al developed a score based on MRI features of the mass, with a sensitivity of 92% and a specificity of 99% for diagnosing autoimmune hypophysitis. This score includes variables such as volume, symmetry, signal intensity, and homogeneity postgadolinium; presence of the posterior pituitary bright spot; stalk size; mucosal swelling; age; and relation to pregnancy. Our patient scored −7 points, indicating autoimmune hypophysitis (score ≤ 0) [10]. Wright et al proposed another score using variables such as diabetes, absence of cavernous sinus invasion, infundibular thickening, and absence of visual symptoms. Our patient met ≥3 criteria, supporting a diagnosis of hypophysitis with an area under the curve of 0.96, sensitivity of 100%, and specificity of 75% [11].

For our patient, additional imaging revealed pulmonary involvement, which helped narrow the differential diagnoses and prompted more targeted laboratory studies and tissue sampling. These studies indicated elevated inflammatory markers and specific antibodies, and our patient met the 2022 American College of Rheumatology criteria for MPA. Although kidney involvement is also typically associated with MPA, the diagnosis of our patient was supported by the presence of anti-MPO antibodies; pulmonary involvement on MRI; and biopsy of the lesion demonstrating reactive mesothelial tissue, focal necrosis, and occasional foreign body giant cells, yielding a diagnostic score > 5 [12]. Often patients with hypophysitis do not have other evident systemic involvement at the time of initial presentation, limiting biopsy options. In such scenarios, ANCA positivity becomes very important. Studies have shown that MPO-ANCA positivity carries a high specificity (often >90%) for MPA, although sensitivity can be somewhat lower (typically around 60-80%), helping guide the diagnosis even in the absence of classic renal or other organ involvement [13].

Our case highlights the diagnostic challenges presented by systemic autoimmune diseases, especially when patients present with rare manifestations such as pituitary involvement in MPA. This case required extensive evaluation involving a multidisciplinary team approach of multiple specialties, including neurology, pulmonology, rheumatology, neuroradiology, and neuroendocrinology. Given the unusual presentation of MPA in this patient, there is a clear need for additional research to extend our understanding of the pathophysiological connections between autoimmune processes and its effects, albeit rare, on the pituitary gland. More detailed studies are needed to develop refined screening and diagnostic criteria, which are vital for ensuring precise diagnoses to enable prompt and optimal treatment to be instituted to achieve good outcomes.

## Learning Points

- Pituitary involvement is rare in both GPA and MPA, but it is particularly more uncommon in MPA. This case emphasizes the need to consider vasculitic etiologies, such as MPA, even when pituitary involvement is observed.
- The overlap in clinical presentations between vasculitic syndromes like GPA and MPA can make the diagnosis challenging, especially when uncommon manifestations like pituitary enlargement are present. Comprehensive imaging and laboratory work, including ANCA testing, are critical in distinguishing these conditions.
- The rarity of pituitary involvement in MPA and lack of specific treatment protocols underscore the need for further research to better understand the pathophysiology and optimize management strategies for this condition.

## Contributors

All authors contributed significantly to the manuscript. K.C.J.Y. supervised the work, reviewed the content, and edited the text. M.R. was responsible for the diagnosis and management of the case. M.M.G. handled manuscript preparation and submission. J.H. provided his neuroradiologic expertise, analyzing and interpreting imaging. S.K. contributed her rheumatologic expertise in the field of vasculitis. All authors reviewed and approved the final draft.

## Data Availability

Data sharing is not applicable to this article as no datasets were generated or analyzed during the current study.
